# A genome-wide association study and genetic risk score for age at diabetes diagnosis support beta-cell dysfunction in young-onset diabetes in Chinese

**DOI:** 10.1186/s13073-026-01760-5

**Published:** 2026-11-12

**Authors:** Baoqi Fan, Claudia H. T. Tam, Andrea O. Y. Luk, Eric S. H. Lau, Cadmon K. P. Lim, Mai Shi, Raymond Wan, Stephanie H. M. Cheung, Yingnan Fan, Alex C. W. Ng, Hongjiang Wu, Aimin Yang, Juliana N. M. Lui, Heung-Man Lee, Elaine Chow, Alice P. S. Kong, Risa Ozaki, Chun-Kwun O, Gechang Yu, Chuiguo Huang, Maggie H. T. Wang, Samuel Y. S. Wong, Eric K. P. Lee, Alfred Hu, Dandan Yan, Weiping Jia, Ka-Fai Lee, Shing-Chung Siu, Grace Hui, Chiu-Chi Tsang, Kam-Piu Lau, Jenny Y. Y. Leung, Elaine Y. N. Cheung, Man Wo Tsang, Grace Kam, Ip-Tim Lau, June K. Y. Li, Vincent T. F. Yeung, Emmy Lau, Stanley Lo, Samuel Fung, Yuk Lun Cheng, Chun-Chung Chow, Xiaodan Fan, Ting-Fung Chan, Kevin Y. L. Yip, Si Lok, Weichuan Yu, Stephen K. W. Tsui, Hui-Yao Lan, Cheuk-Chun Szeto, Nelson L. S. Tang, Brian Tomlinson, Yu Huang, Wing-Yee So, Ronald C. W. Ma, Juliana C. N. Chan

**Affiliations:** 1 Department of Medicine and Therapeutics, Faculty of Medicine, The Chinese University of Hong Kong, Prince of Wales Hospital, Shatin, Hong Kong, China; 2 Li Ka Shing Institute of Health Sciences, Faculty of Medicine, The Chinese University of Hong Kong, Prince of Wales Hospital, Shatin, Hong Kong, China; 3 CUHK-SJTU Joint Research Centre in Diabetes Genomics and Precision Medicine, Hong Kong Institute of Diabetes and Obesity, The Chinese University of Hong Kong, Prince of Wales Hospital, Shatin, Hong Kong, China; 4 The University of Manchester, Manchester, England, UK; 5 Jockey Club School of Public Health and Primary Care, Faculty of Medicine, The Chinese University of Hong Kong, Prince of Wales Hospital, Shatin, Hong Kong, China; 6 Shanghai Sixth People Hospital, Shanghai Jiao Tong University School of Medicine, Shanghai, China; 7 Department of Medicine and Geriatrics, Kwong Wah Hospital, Hong Kong, China; 8 Diabetes Centre, Tung Wah Eastern Hospital, Hong Kong, China; 9 Diabetes and Education Centre, Alice Ho Miu Ling Nethersole Hospital, Hong Kong, China; 10 North District Hospital, Hong Kong, China; 11 Department of Medicine and Geriatrics, Ruttonjee Hospital, Hong Kong, China; 12 Department of Medicine and Geriatrics, United Christian Hospital, Hong Kong, China; 13 Tseung Kwan O Hospital, Hong Kong, China; 14 Department of Medicine, Yan Chai Hospital, Hong Kong, China; 15 Centre for Diabetes Education and Management, Our Lady of Maryknoll Hospital, Hong Kong, China; 16 Department of Medicine, Pamela Youde Nethersole Eastern Hospital, Hong Kong, China; 17 Department of Medicine and Geriatrics, Princess Margaret Hospital, Hong Kong, China; 18 Department of Medicine, Alice Ho Miu Ling Nethersole Hospital, Hong Kong, China; 19 Department of Statistics and Data Science, The Chinese University of Hong Kong, Hong Kong, China; 20 School of Life Sciences, The Chinese University of Hong Kong, Hong Kong, China; 21 Department of Computer Science and Engineering, The Chinese University of Hong Kong, Hong Kong, China; 22 Centre for Applied Genomics, The Hospital for Sick Children, Toronto, Canada; 23 Department of Electronic and Computer Engineering, The Hong Kong University of Science and Technology, Hong Kong, China; 24 School of Biomedical Sciences, Faculty of Medicine, The Chinese University of Hong Kong, Hong Kong, China; 25 Department of Chemical Pathology, Faculty of Medicine, The Chinese University of Hong Kong, Hong Kong, China; 26 School of Medicine, The Chinese University of Hong Kong - Shenzhen, Shenzhen, China; 27 Department of Biomedical Sciences, The City University of Hong Kong, Hong Kong, China

**Keywords:** Age at diabetes diagnosis, Young-onset diabetes, Genome-wide association studies, Genetics in Chinese people

## Abstract

**Background:**

We investigated genetic variations driving young age-at-diabetes-diagnosis (ADD) and developed a genetic risk score (GRS) to identify Chinese individuals at risk of young-onset diabetes (YOD with ADD<40).

**Methods:**

We performed genome-wide association studies (GWAS) for ADD with internal and external replication in 22,611 individuals with type 2 diabetes (T2D, 20% with YOD) and identified Chinese-prioritized loci by conditioning on the polygenic risk score (PRS) of ADD constructed from publicly available datasets. We constructed ADD-GRS using genome-wide independent variants (p<5E-08) and examined its associations with glycemic traits in diabetes and non-diabetes cohorts.

**Results:**

We identified three independent signals at genome-wide significance (*P* < 5×10−8) mapping to *CDKAL1* (rs2206734, β=-0.75 (SE:0.13) per copy of T allele, effect allele frequency (EAF)=0.38, *p*=2.12E-09), *PAX4* (rs61160304, β=-1.01 (SE:0.17) per copy of T allele, EAF=0.19, *p*=2.51E-09), and *KCNQ1* (rs2299620, β=-0.77 (SE:0.14) per copy of G allele, EAF=0.70, p=2.80E-08). The variants at PAX4 and KCNQ1 were independent of the genetic liability estimated by ADD-PRS from Europeans and South Indians. In the diabetes cohort, the ADD-GRS was associated with younger ADD, lower C-peptide, higher HbA1c and pathway-specific T2D-PRS for beta-cell+proinsulin and residual glycemia. Using YOD versus later-onset diabetes as a secondary outcome, *LINC02783/PADI1* (rs72895704, OR:1.52 per copy of A allele, EAF=0.02, *p*=1.93E-06) and *TARS2P1* (rs145218437, OR: 1.73 per copy of C allele, EAF=0.02, *p*=7.57E-08) showed suggestive significance.

**Conclusions:**

Both known and novel T2D loci contribute to ADD in Chinese people and may be used to detect high risk individuals for early intervention to prevent or delay onset of YOD.

**Supplementary Information:**

The online version contains supplementary material available at https://doi.org/10.1186/s13073-026-01760-5.

## Background

The prevalence of young-onset diabetes (YOD) with age-at-diabetes-diagnosis (ADD) less than 40 in Asia was twice that in Europe [1] and affected one in five Asian adults with type 2 diabetes (T2D) [2]. Compared to later-onset diabetes (LOD with ADD after 40), YOD was characterised by lower beta-cell function [3], rapid glycemic deterioration and high lifetime risks of physical and mental illnesses with premature mortality [2, 4–6]. Missed diagnosis, misclassification and suboptimal care contributed to the poor outcomes in YOD calling for more precise diagnosis, prevention and treatment [7].

Type 2 diabetes is characterized by complex interactions amongst genetic, lifecourse and environmental factors with the outcomes influenced by timely diagnosis and quality care [8, 9]. During rapid ecological transition, the Asian phenotype of T2D was characterized by young ADD with low body mass index (BMI) and insufficient insulin secretion to counter insulin resistance, often due to increased visceral adiposity [10]. In a 10-year prospective cohort, a family history of YOD in Chinese conferred 7–9 fold increased risk of T2D versus 2-fold for family history of LOD [11]. In a small case-control cohort of YOD, we first reported the association of YOD with variants in loci (*PAX4*, *CPE*) implicated in islet development and insulin production and secretion with replication in Asians [12, 13]. Hundreds of T2D-loci had now been discovered by genome-wide association studies (GWAS) mainly including older individuals of European descent [14]. In the Progress in Diabetes Genetics in Youth (ProDiGY) Consortium including youths with T2D under the age of 20, mainly of European and African ancestry, seven genome-wide significant loci, including a novel locus in *PHF2* had been discovered [15]. In a trans-ancestry GWAS for ADD, both known and novel loci were identified in South Asians and Europeans [16] with data gaps in East Asians.

In this study, we used genotype data from multiple cohorts to identify genetic variants associated with ADD (continuous) as primary phenotype and YOD (categorical) as secondary phenotype. We developed a genetic risk score (GRS) to evaluate its association with glycemic traits in diabetes and non-diabetes cohorts.

## Methods

### Study participants

We included individuals from multiple cohorts comprising (1) Hong Kong Diabetes Register (HKDR, 1995–2019); (2) Hong Kong Diabetes Biobank (HKDB) and TRansomics ANalysis of Complications ENdpoints in Diabetes (TRANSCEND) Consortium (HKDB, 2014–2019 with 3 phases of genotyping, HKDB-P1,2,3); (3) Precision Medicine to Redefine Insulin Secretion and Monogenic Diabetes-Randomized Controlled Trial (PRISM-RCT, 2020–2024) Cohort with YOD-patients aged less than 50; (4) community-based Shanghai Nicheng Cohort (2013–2014); and (5) non-diabetes cohort confirmed by 75 gram oral glucose tolerance test (OGTT) at baseline or after 10 years of follow-up from the community-based Better Health for Better Hong Kong (BHBHK) Cohort (2002–2014). We excluded patients with physician-diagnosed T1D (diabetic ketoacidosis or continuous insulin requirement within 12 months of diagnosis) or missing data. The final dataset included individuals with T2D from HKDR (*n* = 6,011) [17], HKDB (*n* = 13,176) [18], PRISM (*n* = 873) [19] cohorts. Age at diabetes diagnosis was based on self-reporting. The HKDB cohort were genotyped at three phases [HKDB-P1 (*n* = 5,987), HKDB-P2 (*n* = 5,326), HKDB-P3 (*n* = 1,863)]. We replicated our findings in 2,551 individuals with T2D from Shanghai Nicheng Cohort [20]. We included individuals without diabetes who had genotyping data from the BHBHK (*n* = 652) cohort [21] (Supplementary methods and Figure S1).

### Study design

In **stage one**, we conducted a two-stage GWAS. In stage I (discovery), we performed GWAS separately in HKDR and HKDB-P1 and meta-analyzed results to identify associated signals. In stage Ⅱ (internal replication), we evaluated the lead variants with suggestive *p* value threshold (*p* < 1E-05) from stage I in HKDB-P2 for directionally consistent effects and supportive evidence of association (*p* < 0.05). Lead SNPs were defined as the most significant SNP in each locus, after LD clumping such that selected SNPs were pairwise independent at r^2^ < 0.1 within a ± 250 kb window. This internal replication stage did not apply multiple-testing correction and is interpreted as supportive rather than confirmatory evidence. For lead SNPs showing directionally consistent effects and nominal support in HKDB-P2, we report the pooled effect estimates and standard errors for variants with genome-wide significance (*p* < 5E-08) from the meta-analysis of cohorts HKDR, HKDB-P1 and HKDB-P2 to maximize statistical power and obtain the most precise effect estimates [22]. As a sensitivity analysis, we reported results from a single-stage meta-analysis including all three cohorts [22]. Using similar strategy, we additionally performed GWAS for YOD versus LOD as secondary outcome [22].

Genome-wide independent variants were externally replicated in the Shanghai Nicheng Cohort. We compared the associations of these top signals for (1) YOD using cases (HKDR and HKDB-P2) and controls (individuals without diabetes from BHBHK cohort), and normal-weight (BMI:18.5–23 kg/m^2^) YOD versus obese (BMI ≥ 25 kg/m^2^) LOD from individuals with T2D (HKDR and HKDB cohorts). We performed in-silico replication in trans-ancestry GWAS for ADD [16], GWAS for youth-onset diabetes from ProDigy consortium [15], GWAS for T2D in East Asians (AGEN and DIAMANTE-Asian) [23], Diabetic Cohort-Singapore Prospective Study-SEED GWAS (https://blog.nus.edu.sg/sphs/; https://www.snec.com.sg/research-innovation/key-programme-singapore-epidemiology-of-eye-diseases), and GWAS for T2D in Europeans (DIAMANTE-European) [24].

We investigated secondary independent genetic variants by conditioning on the top variants discovered in the primary analyses. We used the same procedures and criteria in the primary analysis to define genome-wide significant variants to identify secondary loci. We additionally performed conditional analyses to identify Chinese-prioritized loci independent of genetic liability of ADD estimated using data from publicly available trans-ancestry ADD-PRS, South Asian ADD-PRS, and European ADD-PRS; and ADD loci independent of genetic liability for general T2D as estimated by East Asian T2D-PRS (sub-section of “construction of polygenic risk score ”).

**Fig. 1:**
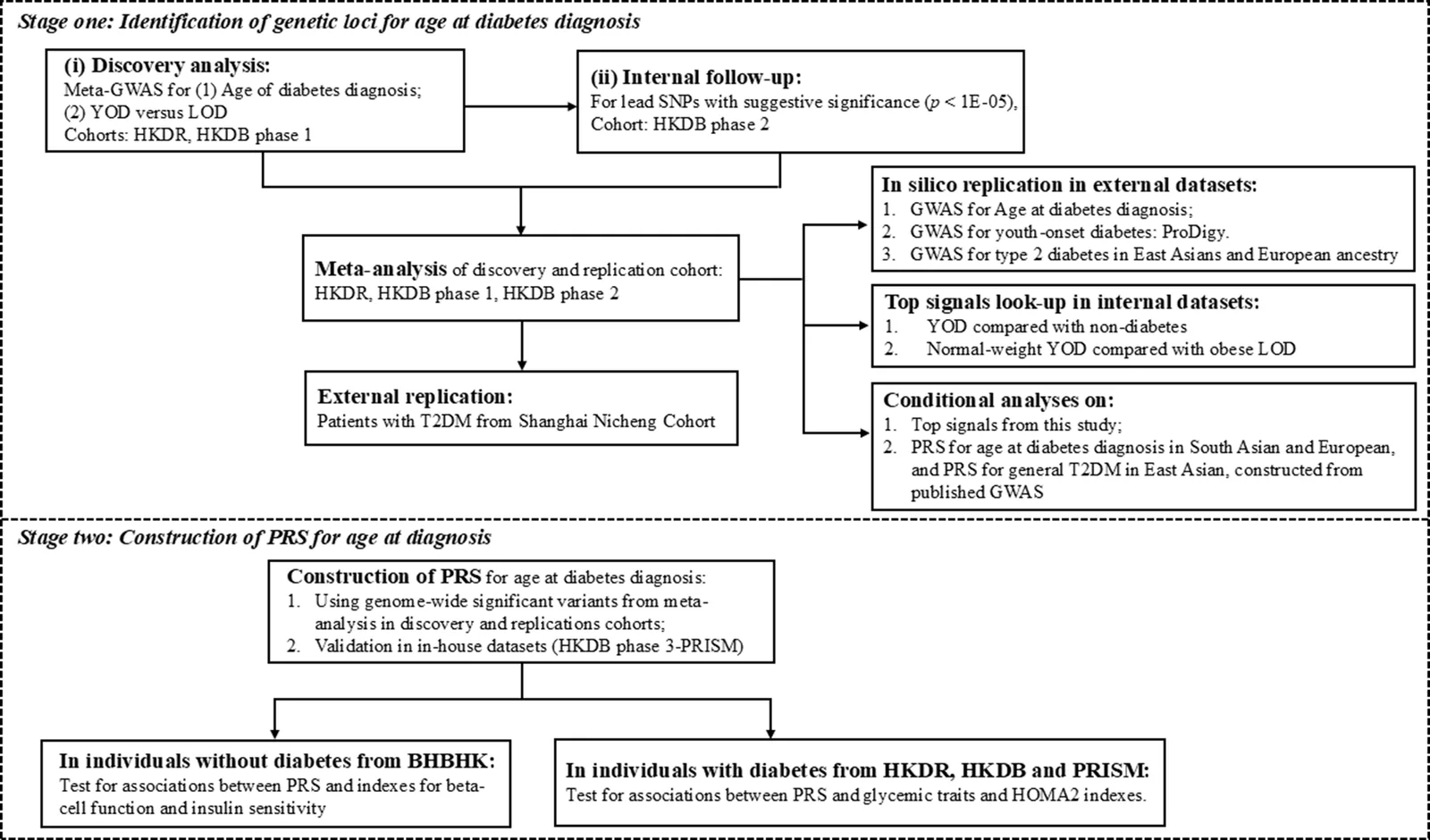
Study design and workflow of genome-wide association studies (GWAS) of age at diabetes diagnosis and related traits in Chinese population with replication in multiple in-house and publicly available databases. In stage one, we performed meta-analysis of genome-wide association studies on individuals with diabetes from discovery cohort combining HKDR and HKDB phase one cohort. Then, we replicated variants with meta-analysis *p* < 1E-05 in internal replication cohort. Top signals from meta-analysis of the combined discovery and internal replication cohorts were looked up in external Chinese cohort and publicly available GWAS summary statistics from different ethnic groups as external validation. We performed conditional analyses to identify secondary signals and Chinese-prioritized loci for YOD. In stage two, we used top signals and computed polygenic risk scores to evaluate their associations with glycemic traits, beta-cell function and insulin resistance in individuals with and without diabetes

In **stage two**, we constructed GRS for ADD (Chinese ADD-GRS) using independent top variants (*p* < 5E-08, LD r^2^ < 0.1) from stage one and validated its performance in the combined cohort of HKDB-P3 and PRISM. We evaluated the associations of top signals and ADD-GRS with glycemic traits and indices of beta-cell function and insulin sensitivity in both diabetes and non-diabetes cohorts **(**Fig. 1, supplementary methods). We adopted the STrengtheningthe REportingof Genetic Association Studies (STREGA) guideline for reporting [25].

### Genotyping, quality control (QC) and imputation

The genotyping arrays for Hong Kong cohorts were: Illumina Omni2.5 + Exome Array for HKDR (*n* = 6011) and 332 individuals without diabetes from BHBHK; Infinium^®^ Global Screening Array for HKDB-P1 (*n* = 5987) and Infinium^®^ Asian Screening Array for HKDB-P2 (*n* = 5326), HKDB-P3 (*n* = 1863), PRISM (*n* = 873) and 320 individuals without diabetes from BHBHK. We applied the same standard quality control (QC) procedures for all genotyping data (supplementary methods). We imputed each batch of genotyping data with the 1000 Genomes Project phase III reference panel (October 2014) using the Michigan Imputation Server. Single nucleotide polymorphisms (SNPs) with minor allele frequency (MAF) < 1% or imputation quality score r^2^ < 0.5 were removed. Genotyping in the Shanghai Nicheng Cohort was performed with Infinium Asian Screening Array and Multi-Ethnic Global Array platform (Illumina, San Diego, USA). Standard QC procedure of Shanghai Nicheng Cohort was reported in supplementary methods.

### Construction of polygenic risk score

A PRS was derived as the sum of doses of risk alleles weighted by the corresponding effect sizes from *β*_*1*_* x*_*1*_ + *β*_*2*_* x*_*2*_ + … + *β*_*k*_*x*_*k*_ + … + *β*_*n*_*x*_*n*_, where *β*_*k*_ was the per-allele effect size for outcome associated with SNP *k*, *x*_*k*_ was the number of effect alleles at SNP *k*, and *n* was the total number of SNPs included in the PRS (from public databases) or GRS (discoveries from this study). We computed unweighted PRS/GRS by setting *β*_*k*_ as integer “1”, and computed weighted PRS/GRS by assigning *β*_*k*_ as the per-allele effect size from summary statistics in each specific analysis. In this study, we calculated four types of PRS/GRS. Firstly, we included lead SNPs (r^2^ < 0.1) with genome-wide significance (*p* < 5E-08) from meta-GWAS of ADD in stage one to compute the Chinese ADD-GRS with the effect alleles and effect sizes of SNPs shown in Table S1. We coded effect allele of each SNP as the allele associated with younger ADD. Thus, the higher ADD-GRS value was associated with younger ADD and higher risk of YOD. Secondly, we used summary statistics from published GWAS to calculate trans-ancestry, South Asian, and European ADD-PRS (effect allele and effect size shown in Table S2) [16]. We replaced missing SNPs in our datasets with proxy SNPs with LD statistics r^2^ > 0.9 to construct the PRS. These PRS were used in the conditional analyses to identify Chinese-prioritized loci independent of genetic liability of ADD estimated by non-Chinese PRS. Thirdly, we constructed the East Asian T2D-PRS based on 265 variants from AGEN T2D Consortium [23] using genotype data available in our datasets with effect alleles and effect sizes shown in Table S3. This PRS was used in the conditional analysis to identify ADD loci independent of genetic liability of general T2D estimated by T2D-PRS from East Asians. Finally, we used multi-ancestry hard-clustering T2D genetic clusters to construct the eight pathway-specific PRS (pwPRS) for T2D with East Asian-specific effect size [26, 27]. We constructed a total T2D-PRS using genome-wide independent SNPs (*n* = 1289) reported by Suzuki et al. weighted by East Asian-specific effect size [26]. We examined the correlations between these PRS and ADD-GRS computed using the top SNPs in this study. The SNPs included in the total T2D-PRS and each cluster had been reported [27].

### Statistical analysis

Data are presented as mean ± SD or median (interquartile range, IQR). For comparison analyses, Student’s t-test and Analysis of Variance were used for normally-distributed variables, Mann–Whitney U test for skewed variables, Chi‐square (χ^2^) and Fisher’s exact test for categorical variables. Age at diabetes diagnosis (in years) was analyzed as a continuous outcome. For each cohort, we performed GWAS under an additive genetic model, fitting a linear regression of ADD on SNP dosage adjusted for sex, BMI and the first four principal components (PCs):

$$\eqalign{& \>{\rm{AD}}{{\rm{D}}_i} = \beta {\>_0} + \beta {\>_g}{G_i} + \beta {\>_{{\rm{sex}}}}{\rm{se}}{{\rm{x}}_{\rm{i}}} \cr & \,\,\,{\rm{ + }}\,\beta {\>_{{\rm{BMI}}}}{\rm{BM}}{{\rm{I}}_{\rm{i}}}{\rm{ + }}\sum\limits_{{\rm{k = 1}}}^4 {\beta {\>_{\rm{k}}}{\rm{P}}{{\rm{C}}_{{\rm{ik}}}}{\rm{ + }}{\epsilon _{\rm{i}}},} \cr} $$

where $\:{G}_{i}$ is the imputed allele dosage (0–2) for the effect allele, and $\:{\epsilon}_{i}$is the residual error. Covariates were variance-standardized. We then meta-analyzed cohort-specific GWAS summary statistics using GWAMA under a fixed-effect model, after excluding variants with evidence of heterogeneity [28]. In a sensitivity analysis, we performed an overall meta-GWAS for ADD in HKDR, HKDB-P1 and HKDB-P2, to mitigate reduced sensitivity for missing variants that did not pass the intermediate threshold. As a secondary outcome, we used similar strategy and performed GWAS for YOD versus LOD under an additive genetic model by fitting a logistic regression on SNP dosage, adjusted for sex, BMI and the first four PCs. Odds ratios with 95% CI were reported.

We illustrated the results by Manhattan plots and quantile-quantile plots using the FUMA online tools [29]. Regional plots around genome-wide loci (± 100 kb of the top variant) were generated using LocusZoom (http://locuszoom.org/). We performed additional sensitivity analyses to examine the robustness of results by (1) conducting GWAS in individuals with T2D after excluding those with known mutations in monogenic diabetes genes; and (2) testing associations of top SNPs with ADD in individuals with T2D who were diagnosed within three years before assessment to reduce recall bias of self-reported ADD. Unweighted and weighted PRS were transformed to z score in each cohort, or stratified by quintiles of the raw score values for subsequent analyses (supplementary materials). In each model, we adjusted for covariates which were explained in the footnotes, methods section of supplementary materials, and statements in the results section. P-values were adjusted for multiple comparisons using the Benjamini–Hochberg false discovery rate (FDR), with q < 0.05 considered significant. Population attributable fraction (PAF, 95% CI) of ADD risk alleles for YOD among individuals with T2D was calculated using the frequency and effect size of the implicated risk allele (supplementary methods). All analyses were performed using PLINK v1.9 (https://www.cog-genomics.org/plink/1.9/) and v2.0 (https://www.cog-genomics.org/plink/2.0/)[31], GWAMA [28], and R version 4.3.1. Supplementary materials show details of baseline assessments, definitions of outcomes and functional annotations of variants.

## Results

We included 6,011 individuals with T2D from HKDR (age: 57.4 ± 13.3 years, 45.3% men; ADD: 49.9 ± 12.7 years, 22.0% YOD), 13,176 individuals with T2D from HKDB (age: 60.9 ± 11.2 years, 59.4% men, ADD: 50.0 ± 11.3 years, 17.7% YOD), 873 individuals with T2D from the PRISM cohort (age: 40.7 ± 6.5 years, 56.7% men, ADD: 32.5 ± 6.7 years, 89.1% YOD), 2,551 individuals with T2D from Shanghai Nicheng Cohort (age: 58.6 ± 6.9 years, 40.9% men, ADD: 56.6[50.6, 61.6] years, 2.0% YOD), and 652 individuals without diabetes from BHBHK (age: 45.2 ± 11.4 years, 48.7% men).

### Meta-analysis of GWAS for age-at-diabetes-diagnosis

**Fig. 2:**
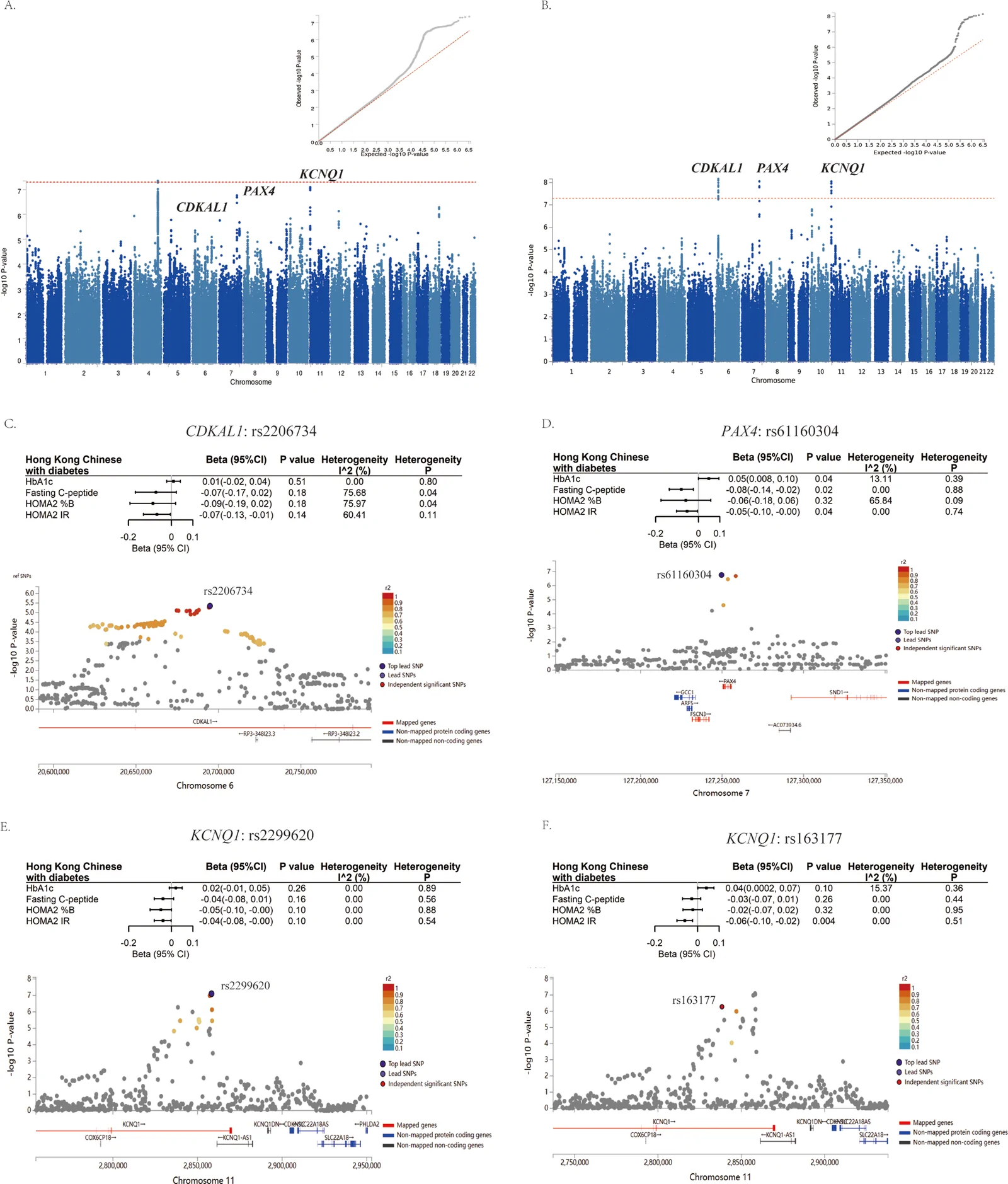
Manhattan plots and Q-Q plots of genome-wide association studies (GWAS) of age at diabetes diagnosis (ADD) in discovery cohort (**A**) and from overall meta-GWAS of combined discovery and internal replication cohort (**B**), regional plots and forest plots for top variants and their corresponding results of associations with glycemic traits in the diabetes cohorts of HKDR, HKDB and PRISM (**C-F**). This figure shows Manhattan plots and Q-Q plots for GWAS of ADD in discovery cohort (**A**) and from overall meta-GWAS of combined discovery and internal replication cohort (**B**), regional plots and associations of HbA1c, fasting C-peptide, HOMA2%B, and HOMA2 IR with top variants identified from GWAS of ADD (**C-F**). The red dashed line indicates the p value of 5E-08. The values of fasting C-peptide, HOMA2%B and HOMA2 IR were logarithmically transformed with base 2. The GWAS was adjusted for sex, BMI and the first four PCs. The associations with glycemic traits were adjusted for age, sex and the first four PCs. *p*-values were adjusted using the Benjamini-Hochberg procedure. Abbreviations: *KCNQ1*, potassium voltage-gated channel subfamily Q member 1; *CDKAL1*, CDK5 regulatory subunit associated protein 1 like1; *PAX4*, paired box 4

We meta-analyzed GWAS on ADD adjusted for BMI including 11,998 individuals with T2D in the discovery cohort (HKDR+HKDB-P1) (baseline characteristics in Table S4) and identified 28 lead SNPs at 28 loci at *p* < 1E-05 (Fig. 2A, Table S5) among 6,625,855 genetic variants with a genomic inflation factor λ_GC_ of 1.042. Three signals at three loci were followed up in HKDB-P2 cohort (0.0001 < *p* < 0.04, Table S5) and reached genome-wide significance (*p* < 5E-08) in the meta-analysis of discovery (HKDR and HKDB-P1) and internal replication (HKDB-P2) cohorts (λ_GC_: 1.057) (Fig. 2B). These loci were *CDKAL1* at chromosome 6 (rs2206734, effect allele frequency (EAF) = 0.38, β=−0.75 (Standard error [SE]:0.13) per copy of T allele, *p* = 2.12E-09), *PAX4* at chromosome 7 (rs61160304, EAF = 0.19, β=−1.01 (SE:0.17) per copy of T allele, *p* = 2.51E-09), and *KCNQ1* at chromosome 11 (rs2299620, EAF = 0.70, β=−0.77 (SE:0.14) per copy of G allele, *p* = 2.80E-08) (Table 1; Fig. 2C-E). The sensitivity meta-GWAS in discovery and internal follow-up cohorts (HKDR, HKDBP1, HKDBP2) identified another lead SNP at *KCNQ1* (rs163177, EAF = 0.48, β=−0.75 (SE: 0.13) per copy of G allele, *p* = 3.58E-09, Table 1, Table S6, Fig. 2F). In the external Shanghai Nicheng Cohort, all four top SNPs showed consistent directions of effect with those observed in the meta-analysis of discovery and internal replication cohorts, but none reached statistical significance under the threshold adjusted for multiple comparisons (Table S5-S6).

Compared to the individuals without diabetes from BHBHK cohort, four signals were associated with YOD [rs2206734 at *CDKAL1*, OR = 1.29 (1.13, 1.48) per copy of T allele, *p* = 1.32E-04; rs61160304 at *PAX4*, OR = 1.57 (1.23, 1.83) per copy of T allele, *p* = 5.73E-06; rs2299620 at *KCNQ1*, OR = 1.54 (1.34, 1.78) per copy of G allele, *p* = 1.06E-09, rs163177 at *KCNQ1*, OR = 1.41 (1.23, 1.61) per copy of G allele, *p* = 8.34E-07, Table 1, baseline characteristics in Table S7]. These signals were also associated with normal-weight-YOD versus obese-LOD [rs2206734 at *CDKAL1*, OR = 1.30 (1.18, 1.45) per copy of T allele, *p* = 4.56E-07; rs61160304 at *PAX4*, OR = 1.30 (1.13, 1.49) per copy of T allele, *p* = 1.52E-04; rs2299620 at *KCNQ1*, OR = 1.19 (1.06, 1.34) per copy of G allele, *p* = 0.003; rs163177 at *KCNQ1*, OR = 1.19 (1.07, 1.32) per copy of G allele, *p* = 0.001, baseline characteristics in Table S8].

In in-silico replication analysis using externally-available GWAS, none of these variants was associated with ADD in Europeans or South Asians from UK Biobank (Table S9). The SNP rs9368219, in LD with the top SNP at *CDKAL1*, was associated with ADD in non-Chinese cohorts (Table 1). This SNP had larger effect size in South Asians (β=−0.92) than Chinese (β=−0.75) or mixed populations (β=−0.06). Except for *PAX4* which was nearly monomorphic in Europeans, the other three variants were associated with BMI-adjusted T2D at suggestive genome-wide significance in East Asians and Europeans (Table 1). The two variants at *KCNQ1* were associated with youth-onset diabetes in the multi-ethnic ProDiGY Consortium (OR:1.04 to 1.06, Table 1). The EAF of variant at *CDKAL1* was 0.38 in Chinese and 0.18 in Europeans with similar EAF for variants at *KCNQ1* in both populations **(**Table 1**).** Among individuals with diabetes, the risk ratio (RR)-based population attributable fraction (PAF) of YOD contributed by top signals ranged from 4.6 (2.4, 6.8)% (rs61160304 at *PAX4*) to 14.2 (8.0, 20.1)% (rs2299620 at *KCNQ1*), estimated under additive genetic model and assuming Hardy-Weinberg Equilibrium (Table S10).

### Meta-analysis of GWAS for YOD versus LOD

The discovery analysis for YOD (λ_GC_:1.018) identified 18 lead variants at 18 loci with *p* < 1E-05 (Figure S2A, Table S11, baseline characteristics in Table S12). In the HKDB-P2, rs72895704, located intergenically between *LINC02783 and PADI1*, was marginally replicated [OR:1.32 (0.96, 1.83) per copy of A allele, *p* = 0.089]. In the meta-analysis of discovery and replication cohorts (λ_GC_:1.011), rs72895704 (EAF = 0.02) was associated with YOD versus LOD at suggestive significance with an OR of 1.52 (1.28, 1.80, *p* = 1.93E-06) (Table 1, Figure S2B). The sensitivity meta-GWAS of discovery and internal replication cohorts identified another lead variant located at *TARS2P1* with near genome-wide significance (rs145218437, OR: 1.73 per copy of C allele, EAF = 0.02, *p* = 7.57E-08, Table 1, Table S13, Figure S2C-S2D). These two variants were not replicated in the external Shanghai Nicheng Cohort [rs72895704: OR: 2.82 (1.57, 4.57), *p* = 0.103; rs145218437: OR: 0.88 (0.28, 2.75), *p* = 0.820; baseline characteristics in Table S14)]. SNP rs72895704 located intergenically between *LINC02783* and *PADI1* was associated with ADD in South Asians from UK Biobank (β=−0.16 (SE:0.08), *p* = 0.043, Table S9). It was also associated with youth-onset diabetes in the multi-ethnic ProDiGY Consortium (OR:1.02, *p* = 0.03, Table 1), where the association was driven by Hispanic ancestry (OR: 1.12, *p* = 2.19E-04) but not by Europeans (OR: 0.98, *p* = 0.592, Table 1). The RR-based PAF for YOD in diabetes population were 1.7 (0.7, 2.7)% for rs72895704 and 1.6 (0.9, 2.4)% for rs145218437 (Table S10). In the combined diabetes cohorts, the SNP-based heritability (h^2^) for ADD was 0.16 (SE: 0.03) and 0.15 (0.06) for YOD.

**Table 1:** Top signals from genome-wide associations study (GWAS) of age at diabetes diagnosis and their associations with related traits in in-house Chinese population and publicly-available GWAS data from other Consortiums with Asian or non-Asian individuals

SNP	CHR	POS	EA/ NEA	EAF	Gene	Effect size	*P* value	Traits	Sample size	Ethnics	Data source	Ref.
Primary phenotype: Age at diabetes diagnosis												
rs2206734^a, b^	6	20,694,884	T/C	0.38	*CDKAL1*	-0.75	2.12E-09	Age at diabetes diagnosis	17,327 individuals with T2D	Hong Kong Chinese	Hong Kong Chinese	
				0.38		1.29^†^	1.32E-04	YOD versus non-diabetes	2,351 T2D cases and 853 healthy controls	Hong Kong Chinese	Hong Kong Chinese	
				0.37		1.22^†^	5.83E-7	T2D adjusted BMI	10,248 individuals	Mostly East Asians	Diabetic Cohort - Singapore Prospective Study - SEED GWAS	
				0.18		1.17^†^	4.70E-66	T2D adjusted BMI	74,124 T2D cases and 824,006controls	European ancestry	DIAMANTE T2D GWAS (2022)	[30]
rs9368219* tagged to rs2206734	6	20,674,691	T/C	0.26	*CDKAL1*	-0.92	6.59E-08	Age at diabetes diagnosis	9,911 individuals with diabetes	South Asian Indians	the DMDSC cohort and migrated South Asians in the UKBB	[16]
rs9368219* tagged to rs2206734	6	20,674,691	T/C	0.19	*CDKAL1*	-0.06	2.29E-08	Age at diabetes diagnosis	34,001 individuals with diabetes	Multi-ethnics	Transethnic Meta-analysis of DMDSC, GoDARTS, GoSHARE and UKBB	[16]
rs61160304^a, b^	7	127,249,659	T/C	0.19	*PAX4*	-1.01	2.51E-09	Age at diabetes diagnosis	17,327 individuals with T2D	Hong Kong Chinese	Hong Kong Chinese	
				0.19		1.57^†^	5.73E-06	YOD versus non-diabetes	2,351 T2D cases and 853 healthy controls	Hong Kong Chinese	Hong Kong Chinese	
				0.11		1.32^†^	3.28E-123	T2D	77,418 T2D cases and 356,122 healthy controls	East Asians	AGEN and DIAMANTE T2D GWAS	[22]
rs2299620^a^	11	2,858,295	C/T	0.70	*KCNQ1*	-0.77	2.80E-08	Age at diabetes diagnosis	17,327 individuals with T2D	Hong Kong Chinese	Hong Kong Chinese	
				0.70		1.54^†^	1.06E-09	YOD versus non-diabetes	2,351 T2D cases and 853 healthy controls	Hong Kong Chinese	Hong Kong Chinese	
				0.63		1.26^†^	1.25E-06	T2D adjusted BMI	10,248 individuals healthy controls	Mostly East Asians	Diabetic Cohort - Singapore Prospective Study - SEED GWAS	
				0.91		1.25^†^	3.60E-26	T2D adjusted BMI	74,124 T2D cases and 824,006 controls	European ancestry	DIAMANTE T2D GWAS (2022)	[30]
						1.05^†^	2.33E-8	T2D in youth versus adults without diabetes	3,006 cases and 6,061 adult controls	Multi-ethnics	ProDiGY Consortium	[15]
						1.06^†^	5.38E-9	T2D in youth versus adults without diabetes	1,275 cases and 3,559 adult controls	Hispanic ancestry	ProDiGY Consortium	[15]
				0.91		1.06^†^	0.072	T2D in youth versus adults without diabetes	664 cases and 1,434 adult controls	European ancestry	ProDiGY Consortium	[15]
rs163177^b^	11	2,838,413	C/T	0.48	*KCNQ1*	-0.75	3.58E-09	Age at diabetes diagnosis	17,327 individuals with T2D	Hong Kong Chinese	Hong Kong Chinese	
				0.48		1.41^†^	8.34E-07	YOD versus non-diabetes	2,351 T2D cases and 853 healthy controls	Hong Kong Chinese	Hong Kong Chinese	
				0.44		1.17^†^	2.54E-123	T2D	77,418 T2D cases and 356,122 healthy controls	East Asians	AGEN and DIAMANTE T2D GWAS	[22]
				0.50		1.10^†^	1.60E-34	T2D adjusted BMI	74,124 T2D cases and 824,006 controls	European ancestry	DIAMANTE T2D GWAS (2022)	[30]
						1.04^†^	3.02E-8	T2D in youth versus adults without diabetes	3,006 cases and 6,061 adult controls	Multi-ethnics	ProDiGY Consortium	[15]
Secondary phenotype: YOD versus LOD												
rs72895704^a^	1	17,527,269	A/C	0.02	*LINC02783/PADI1*	1.52^†^	1.93E-06	YOD versus LOD	3,652 YOD and 15,538 LOD	Hong Kong Chinese	Hong Kong Chinese	
						1.02^†^	0.03	T2D in youth versus adults without diabetes	3,006 cases and 6,061 adult controls	Multi-ethnics	ProDiGY Consortium	[15]
						1.12^†^	2.19E-04	T2D in youth versus adults without diabetes	1,275 cases and 3,559 adult controls	Hispanic ancestry	ProDiGY Consortium	[15]
				0.06		0.98^†^	0.592	T2D in youth versus adults without diabetes	664 cases and 1,434 adult controls	European ancestry	ProDiGY Consortium	[15]
				0.04		-0.16	0.043	Age at diabetes diagnosis	1,426 individuals with diabetes	South Asians	UK Biobank	
				0.06		1.42^†^	0.002	CKD and DKD adjusted for HbA1c-BMI in T1D	14,039 individuals with T1D	European ancestry	the JDRF Diabetic Nephropathy Collaborative Research Initiative GWAS study	[43]
rs145218437^b^	4	136,590,722	C/T	0.02	*TARS2P1*	1.73^†^	7.57E-08	YOD versus LOD	3,652 YOD and 15,538 LOD	Hong Kong Chinese	Hong Kong Chinese	

Note: (1) SNPs with superscript “*” are in linkage disequilibrium (r^2^ > 0.8) with top SNPs; Effect size with superscript “†” were odds ratios, and others were beta coefficients; (2) SNPs with superscript “a” are the top SNPs from two-stage GWAS; SNPs with superscript “b” are the top SNPs from meta-GWAS of three cohorts (HKDR, HKDB-P1, HKDB-P2) (3). Allele frequency for published GWAS were referred from information in gnomAD (4). Abbreviations: AGEN, the Asian Genetic Epidemiology Network; *CDKAL1*, CDK5 regulatory subunit associated protein 1-like1; CHR, chromosome; DMDSC, Dr Mohan’s Diabetes Specialties Centre; DIAMANTE, the Diabetes Meta-analysis of Trans-ethnic Association Studies; GoDARTS, Genetics of Diabetes Audit and Research in Tayside Scotland (GoDARTS); EA, effect allele; GoSHARE, Genetics of Scottish Health Research Register; JDRF, Juvenile Diabetes Research Foundation; *KCNQ1*, potassium voltage-gated channel subfamily Q member 1; NEA, non-effect allele; *PAX4*, paired box 4; POS, position; SNP, single nucleotide polymorphism; *PADI1*, peptidyl arginine deiminase 1; UKBB, the UK Biobank; ProDiGY, the Progress in Diabetes Genetics in Youth; T2D adj BMI, type 2 diabetes adjusted for body mass index; *TARS2P1*, threonyl-tRNA synthetase 2 pseudogene 1; (5) YOD was defined as diabetes diagnosed before the age of 40. T2D in youth from ProDiGY consortium refers to youths with diabetes before the age of 20

### Conditional analyses

On conditional analysis, we did not identify additional genome-wide significant variants. We constructed trans-ancestry ADD-PRS, South Asian ADD-PRS, European ADD-PRS and East Asian T2D-PRS from published GWAS summary statistics. They were included as covariates in meta-GWAS in the combined discovery and internal replication cohorts. Only East Asian T2D-PRS and South Asian ADD-PRS were associated with ADD in our Chinese cohorts (Table S15). When conditioned on East Asian T2D-PRS, all four top signals were rendered non-significant (Figure S3D). The top signals at *CDKAL1* locus were rendered non-significant with conditioning on South Asian ADD-PRS. The three top loci remained genome-wide significant when conditioned on European ADD-PRS or trans-ancestry ADD-PRS (Figure S3A-S3C).

### Functional annotations

We did not identify expression quantitative trait loci using the three top signals for ADD and the two for YOD, except for rs163177 at *KCNQ1*. This SNP was associated with expression level of *TNNI2* (troponin I2, fast skeletal type) in basal ganglia of the brain (Table S16). We did not observe significant signals from gene set enrichment analysis and KEGG pathway analysis after correction for multiple comparisons (Table S17-S18).

### Top variants and GRS with metabolic traits in diabetes and non-diabetes cohorts

In the non-diabetes cohort, per risk allele of rs2299620 at *KCNQ1* was associated with decreased insulinogenic index (β=−0.21 (−0.37, −0.05), *p* = 0.03) and increased Matsuda index (β = 0.13 (0.02, 0.25), *p* = 0.04) (Table S19). In the diabetes cohort, rs61160304 located near *PAX4* was associated with higher HbA1c (β = 0.05 (0.008, 0.10), *p* = 0.04) and lower fasting C-peptide (β=−0.08 (−0.14, −0.02), *p* = 0.02, Fig. 2D, Table S20). The risk allele of rs163177 at *KCNQ1* was associated with lower HOMA2-IR (β=−0.06 (−0.10, −0.02), *p* = 0.004, Fig. 2E). These associations remained significant after controlling FDR.

**Fig. 3:**
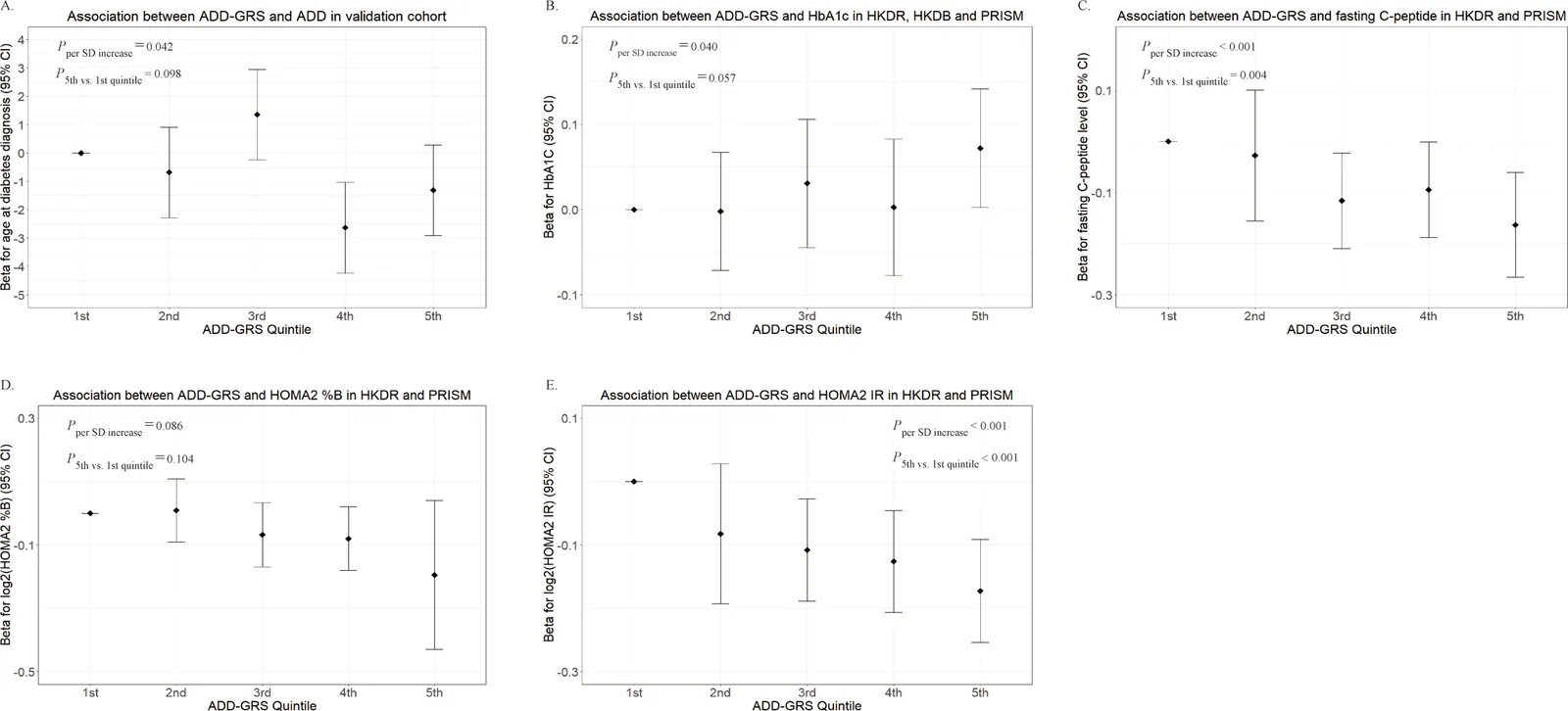
Bar charts of validation for ADD-GRS (**A**) and its associations with HbA1c (**B**), fasting C-peptide (**C**), HOMA2%B (**D**) and HOMA2 IR (**E**) in diabetes. Panel (**A**) was for association between ADD-GRS and age at diabetes diagnosis in validation cohort of individuals with T2D from HKDB-P3 and PRISM, with adjustment of sex and the first four principal components (PCs). Panel (**B**) were meta-analyzed in individuals with T2D from HKDR, HKDB-P1, HKDB-P2, HKDB-P3 and PRISM with adjustment of age at assessment, sex and the first four PCs. Panel (**C-E**) were meta-analyzed in individuals with T2D from HKDR and PRISM with adjustment of age at assessment, sex and the first four PCs. *P*_per SD increase_ indicates *p* values for associations with per 1-SD increase of standardized PRS. *P*_5th vs. 1st quintile_ indicates *p* values for associations with top 20% of the PRS compared with the bottom 20% of the PRS. All *p* values were adjusted for multiple comparisons by the Benjamini-Hochberg procedure

We constructed a Chinese ADD-GRS using three genome-wide independent variants (r^2^ < 0.1) identified from the two-stage GWAS for ADD. Higher level of ADD-GRS was associated with younger ADD. Per-SD increase of unweighted and weighted GRS was associated with 0.54 (*p* = 0.038) and 0.53 (*p* = 0.042) years younger for ADD respectively in validation cohort of HKDB-P3 and PRISM (Fig. 3, Table S21-S22). In the diabetes cohort, increased ADD-GRS was associated with lower log_2_-C-peptide (per-SD increase: β=−0.06 (−0.09, -0.03), *p* < 0.001; top 20% vs. bottom 20%: β=−0.16 (−0.27, -0.06), *p* = 0.002) and higher HbA1c (per-SD increase: β = 0.03 (0.003, 0.05), *p* = 0.040; top 20% vs. bottom 20%: β = 0.07 (0.002, 0.14), *p* = 0.043, Fig. 3, Table S23) after controlling for multiple comparisons and covariates of age at assessment, sex and the first four PCs. The ADD-GRS was correlated with the overall, beta-cell + PI (proinsulin), and residual glycaemic T2D pwPRS constructed from top loci identified by multi-ancestry meta-GWAS (Figure S4). In the non-diabetes cohort, there was no association between ADD-GRS and one-hour plasma glucose, beta-cell function or insulin sensitivity indices during five-point OGTT (Table S24).

### Secondary analyses

We performed sex-specific analyses. In men, a SNP rs12190327 located near *OLIG3* (oligodendrocyte transcription factor 3) at chromosome 6 reached near genome-wide significance (β=−2.86 (0.53) per copy of A allele, *p* = 7.69E-08) with an EAF of 0.05 (Figure S5A). This SNP was replicated in HKDB-P2 (β=−1.30 (SE:0.65) per copy of A allele, *p* = 0.046) with borderline genome-wide significance in meta-GWAS of the discovery and internal replication cohort (β=−2.24 (SE:0.41) per copy of A allele, *p* = 5.75E-08, Table S25). There was no genome-wide significant signal in women when analysed for ADD or YOD versus LOD in two-stage GWAS (Figure S5, Table S26).

In the HKDR, 1021 patients with YOD had targeted exome sequencing of monogenic diabetes genes [32]. We repeated the meta-GWAS after excluding 20 individuals with pathogenic/likely pathogenic variants, and the top signals for ADD or YOD remained significant (Table S27-S28, Figure S6). To reduce recall bias from self-reported phenotype of ADD, we restricted analyses in individuals with T2D diagnosed within three years, where the top signals remained associated with ADD (Table S29).

## Discussion

The aggressive clinical course of YOD especially in non-Europeans, calls for better understanding of its causality. In this first and largest GWAS of ADD including 22,611 Chinese individuals with T2D, we identified three genome-wide independent variants located at or near three published T2D risk loci from the two-stage GWAS: *KCNQ1*, *PAX4*, and *CDKAL1*. The increased ADD-GRS was associated with younger ADD, lower fasting C-peptide, and higher HbA1c in diabetes cohort. The EAFs of these variants ranged from 0.19 to 0.70 with beta-coefficients for ADD ranging from −1.01 to -0.75. Each of these three SNPs contributes to an attributable risk of 4.6–14.2% for YOD in individuals with diabetes. Our results highlighted inter-ethnic differences in EAF and effect size for the same variant, emphasizing the importance of multi-ethnic studies to unravel the genetic background of development of diabetes at young age as an emerging global concern [1]. These results also filled an important knowledge gap on genetic predisposition of YOD in Chinese. Differences in genome architecture as well as ancestral, cultural, ecological and other factors might contribute to inter-ethnic variations in disease trajectories, treatment responses and clinical outcomes, despite being exposed to similar external stressors [33]. The heavier genetic loading in YOD versus LOD suggested that these genetic markers might be used to identify high-risk individuals for early intervention [34].

Amongst the three loci, *KCNQ1* and *CDKAL1* have been reported in candidate gene analysis and multi-ethic GWAS for T2D [23, 35]. In an earlier GWAS including Chinese patients mainly with YOD, we first reported the association of rs10229583 near *PAX4* with T2D and ADD (β_unadjusted_ ± SE = − 0.90 ± 0.24, *p* = 2.3 × 10^− 4^) with replication in Chinese from Shanghai [36]. This SNP was not in LD with rs61160304 discovered in this study. In a trans-ethnic meta-analysis of whole exome sequencing data including data from our cohorts, *PAX4* was one of the most prominent East Asian specific loci for T2D [37].

The complexity of T2D can be due to different causes with each cause consisting of different components with each component not being sufficient to cause disease [38]. Screening for variants of ADD or YOD would enable identification of genetically-predisposed individuals with improved precision for mitigating modifiable risk factors, such as obesity. Implementation studies are needed to confirm the value of this personalized strategy [39].

### Importance of beta-cell dysfunction in Asian populations

The top variants identified in this study were all implicated in beta-cell biology. In our non-diabetes cohort, the top variant at *KCNQ1* was associated with lower insulinogenic index. Other researchers had also reported associations of common variants at *CDKAL1* with impaired beta-cell function [40]. Multiple transcription factors are involved in the programming of progenitor cells for differentiation into endocrine or exocrine lineage, with *PAX4* being the first transcription factor in driving islet and later beta-cell differentiation [41]. During glucose-stimulated insulin secretion, ATP generated from glycolysis and mitochondria oxidative phosphorylation increased the cytoplasmic ATP: ADP ratio to close the ATP-sensitive potassium channel encoded by *KCNQ1* [42]. This activates the opening of voltage-gated calcium channels with influx of calcium (Ca^2+^) ions followed by exocytosis of insulin stored in granules, with *KCNQ1* being a known drug target (sulphonylureas) for T2D [43]. The gene *CDKAL1* belongs to methyl-thiol transferase family implicated in hyper-modification of transfer-RNA during ribosomal protein translation. Abnormal modification of *CDKAL1* can lead to dysregulated translation of the essential Lysine (Lys) residues in pro-insulin resulting in abnormal protein folding, endoplasmic reticulum stress and impaired insulin secretion. Besides, *CDKAL1* might also influence mitochondrial morphology and adipose biology [44].

Recently, our group reported the associations of ADD and YOD with beta-cell pathway gene clusters and variants located in 34 genes related to monogenic diabetes [27, 45]. In this study, we found correlations between ADD-GRS and pwPRS associated with beta-cell dysfunction and residual glycemia. In 2734 Han Chinese with newly-diagnosed T2D and 4041 controls, only PRS related to diabetes (25 SNPs) and beta-cell function (10 SNPs) but not that related to other pathways (14 SNPs) were associated with younger ADD and higher odds of YOD [46]. In 11,678 British Pakistani and Bangladeshi individuals, partitioned polygenic scores for insulin deficiency and lipodystrophy were associated with younger onset of T2D [47]. In Chinese people, the relationship between HOMA-beta decline and disease duration was steeper in YOD than LOD [3].

Apart from insulin deficiency, insulin resistance also contributes to onset of diabetes. In individuals with limited beta-cell reserve due to genetic or non-genetic causes (e.g. low birthweight, poor nutrition), external stressors such as obesity, neurohormonal activation due to poor sleep or psychosocial stress or chronic inflammation, can increase endogenous glucose production and cause excessive demand on beta-cells leading to younger ADD [1, 7, 39]. Although we adjusted for BMI, residual confounding is possible, particularly from visceral adiposity, ectopic fat and related metabolic traits that are imperfectly captured by BMI [48].

### Young-onset diabetes and chronic kidney disease

The suggestive YOD-specific variant rs72895704 located intergenically between *LINC02783* and *PADI1* calls for replication in larger cohorts. This variant was associated with youth-onset diabetes in multi-ancestry GWAS (OR: 1.02, *p* = 0.03 in multi-ancestry; OR: 1.12, *p* = 2.19E-04 in Hispanic ancestry) and chronic kidney disease (CKD) in Type 1 diabetes (T1D) (OR:1.43, *p* = 0.003) [15, 49]. Type 1 diabetes, T2D and CKD are complex diseases due to multiple causes involving multiple pathways. The sharing of common variants amongst noncommunicable diseases including diabetes, cardiovascular disease and cancer have been reported. Glucotoxicity and related downstream processes (e.g., oxidative stress, inflammatory cytokines, growth factors) are shared features in T1D and T2D, which can contribute towards the development of CKD [50]. Given the high risk of CKD in patients with YOD independent of risk factors and disease duration [5], we hypothesize that there might be shared pathways between YOD and CKD through chronic hyperglycemia, although replication and mechanistic studies are needed to elucidate the potential role of *LINC02783/PADI1* in the YOD-CKD link.

### A suggestive variant at TARS2P1

Our study identified an additional locus associated with YOD with near genome-wide significance (*p* = 7.57E-08) near *TARS2P1*, which, to our knowledge, has not been reported in the context of diabetes. *TARS2P1* is a pseudogene with unknown function, and its role in YOD remains unclear. A pseudogene is a DNA sequence that resembles a protein-coding gene but typically cannot produce a functional protein. Pseudogene arises through evolutionary processes. Although some pseudogenes can be transcribed and in rare cases may yield low-level peptide products or exert regulatory effects, their biological functions are often uncertain [51]. Further investigation is needed to elucidate the significance of the variant at *TARS2P1*.

### Ethnic differences in variants associated with ADD

Variations in ethnicity and ancestry, the latter being closely linked to biological connections amongst family members, are important considerations in genetic association studies [52]. Earlier large trans-ethnic GWAS for glycemic traits and T2D did not show heterogeneity amongst cohorts of predominant European ancestry [23, 35, 53]. More recent GWAS data from non-European populations highlighted inter-ethnic differences in disease-loci, lead variants at the same locus and effect sizes for the same variant, with some variants being rare in Europeans but common or having low frequency in East Asians [23]. Even for variants with similar MAFs, there are inter-ethnic differences in their associations with subphenotypes of T2D [23]. To this end, variants of *PAX4* and *KCNQ1* were first reported in East Asians with lower beta-cell function than Europeans [36, 54, 55]. Given the multicausality of T2D and the low attributable risk explained by each variant, a holistic approach including the use of modifiable, non-modifiable and genetic factors is needed to unravel the heterogeneity of causes, trajectories, treatment responses and outcomes in different populations and geographies to inform prevention and treatment strategies [52].

In this study, we identified shared and heterogeneous genetic associations of ADD amongst Chinese, South Asians and Europeans. Although variants at *CDKAL1* were associated with ADD in multi-ethnic populations, their effect sizes and MAFs differed across these major populations. In contrast, the signal at *PAX4* locus was monomorphic in Europeans. We accounted for genetic liability of ADD estimated by ADD-PRS derived from South Asians. Except for the variants at *CDKAL1*, the top variants at *PAX4* and *KCNQ1* remained significant and were considered as prioritized variant for ADD in Chinese people. When analysed by sex, we found a suggestive SNP near *OLIG3* implicated in DNA binding and inflammation. Given the differences in biology associated with age and sex, our findings call for more age and sex-specific genetic association studies to unravel the heterogeneity of T2D.

### Potential utility of genetic risk scores and precision prevention and therapy

Diabetes is a polygenic disease where PRS can be used to improve precision in risk prediction, classification and therapies. In this study, the ADD-GRS was associated with ADD in a validation cohort as a standardized continuous score. These results suggest that the ADD-GRS may help identify individuals with high genetic susceptibility to YOD especially under stress conditions. Given the large number of SNPs included in the multi-ancestry T2D-PRS (constructed from lead variants from Suzuki’s paper), our Chinese ADD-GRS including only three SNPs did not further increase the explained variance for ADD. These SNPs which were discovered in a GWAS from Chinese individuals, may better reflect the population-specific genetic architecture compared with PRS derived primarily from multi-ancestry studies. From an implementation perspective, a small panel of variants offers a lower-cost option for genetic testing and risk stratification than a genome-wide panel. Recent studies have highlighted the importance of including OGTT to detect individuals with diabetes or impaired glucose tolerance (IGT) based on 2-hour plasma glucose value. In addition, 1-hour plasma glucose during OGTT was independently associated with progression of diabetes and complications [56]. Experts also pointed out that most diabetes prevention programs recruited individuals with IGT. Given the vulnerability of individuals at risk of YOD as well as the low levels of awareness amongst young people for chronic diseases, genetic testing followed by OGTT in high-risk individuals may provide an alternative solution to engage these individuals for early intervention although implementation studies are needed to confirm its cost-effectiveness [8, 57]. To this end, evidence suggests that relay of personalized information, including genetic information, may motivate behavioral change [8, 58].

Ameliorating glucotoxicity early to preserve beta-cell function is an important strategy to delay or prevent onset of diabetes and its complications [8]. In a modelling study, we estimated a person diagnosed with YOD at the age of 33 may accrue nearly 100 hospitalization-days by age of 70 [6]. By intensifying control of all risk factors to low targets, the hospitalization days can be reduced by 30%. By delaying the ADD to 50 and 65, the number of hospitalization days can be reduced by 40% and 70% respectively [6]. Insulinogenic drugs such as dipeptidyl peptidase inhibitor (DPP4i) and sulphonylureas are highly effective and affordable glucose lowering drugs widely used in Asians [59, 60]. In Asians with YOD, carriers of these causal variants may benefit preferentially from these drugs as reported in meta-analysis and clinical studies [9, 61–63].

### Strengths and limitations

This is the first and largest GWAS of ADD in Chinese people with internal and external replication in independent cohorts. The differences in EAF and effect sizes between Chinese and non-Chinese people highlighted the ethnic differences on the propensity for YOD [55]. Our study also has limitations. Recall bias, lack of symptoms or delayed diagnosis may contribute to imprecisions in self-reported ADD. This imprecision might attenuate the association, and the reported effect sizes may be conservative. Nevertheless, we have performed sensitivity analysis in individuals diagnosed within three years and found that the top variants were strongly associated with ADD. Future work using electronic health records with confirmed diagnosis dates will help confirm these findings. Since the top loci for ADD are established T2D risk loci, we could not confidently distinguish whether their observed associations with ADD reflected greater overall T2D liability with younger onset or locus-specific “timing” effects that modify disease trajectory independent of baseline susceptibility. However, the motivation of our study is to understand the high prevalence of YOD in Chinese population. To this end, our discoveries confirmed the importance of beta-cell dysfunction in Chinese people, and revealed the heterogeneity of genetic associations with ADD amongst major ethnic groups (Chinese, Europeans and South Indicans). These findings call for larger and more ADD-GWAS from diverse ethnic groups, ideally including analyses conditional on overall T2D polygenic risk, to determine whether loci act as true modifiers of the timing of onset of disease. These analyses will further clarify the potential utility of these prediction tools for precision health and medicine. Finally, the functional significance of these top variants in beta-cell and kidney biology requires further elucidation.

## Conclusions

Young-onset diabetes is a growing concern especially in non-European populations [1]. We identified genome-wide independent variants at three gene loci driving young ADD in Chinese people: *KCNQ1*, *CDKAL1* and *PAX4*. The ADD-GRS calculated by these genome-wide independent variants was associated with reduced beta-cell function and poor glycemia in diabetes cohorts. The suggestive loci *LINC02783/PADI1* and *TARS2P1* for YOD calls for more confirmatory evidence. In Asia, one in five adults diagnosed with T2D had YOD. The ADD-GRS may provide a low-cost solution to identify genetically predisposed individuals for early intervention aimed at preserving beta-cell function to delay onset of diabetes and its complications [34].

## Supplementary Information

Below is the link to the electronic supplementary material.

Supplementary Material 1

Supplementary Material 2

Supplementary Material 3

## Data Availability

Consent had not been obtained from study participants for data sharing in public domain, but summary data are assessable via Zenodo repository: https://doi.org/10.5281/zenodo.21972566.

## Abbreviations

ADD: Age at diabetes diagnosis
BHBHK: Better Health for Better Hong Kong
BMI: Body mass index
*CDKAL1*: CDK5 regulatory subunit associated protein 1-like1
DPP4i: Dipeptidyl peptidase inhibitor
EAF: Effect allele frequency
GWAS: Genome-wide association studies
*KCNQ1*: Potassium voltage-gated channel subfamily Q member 1
LOD: Late-onset diabetes
OGTT: Oral glucose tolerance test
*OLIG3*: Oligodendrocyte transcription factor 3
*PAX4*: Paired box 4
*PADI1*: Peptidyl arginine deiminase 1
PC: Principal component
PRISM-RCT: Precision Medicine to Redefine Insulin Secretion and Monogenic Diabetes-Randomized Controlled Trial
ProDiGY: The Progress in Diabetes Genetics in Youth
PRS: Polygenic risk score
pwPRS: Pathway-specific PRS
SNPs: Single nucleotide polymorphisms
*TARS2P1*: Threonyl-tRNA synthetase 2 pseudogene 1
T1D: Type 1 diabetes
T2D: Type 2 diabetes
TRANSCEND: TRansomics ANalysis of Complications ENdpoints in Diabetes
YOD: Young-onset diabetes
