# Supplementary material for: A genome-wide association study and genetic risk score for age at diabetes diagnosis support beta-cell dysfunction in young-onset diabetes in Chinese
Source: Genome Med. 2026 Nov 12;18:177. doi: 10.1186/s13073-026-01760-5 (PMC13756970; doi:10.1186/s13073-026-01760-5)
Supplement: Supplementary file 1 [file 13073_2026_1760_MOESM1_ESM.docx]

**SUPPLEMENTARY MATERIALS**

**Cohort Description**

***Hong Kong Diabetes Register (HKDR)***

The HKDR was established in 1994 by the Diabetes and Research Care team at CUHK-PWH. Patients with physician-diagnosed diabetes who attended the Diabetes and Endocrine Centre at PWH for diabetes complication screening were consecutively recruited into HKDR. Ethical approval was obtained from the Clinical Research Ethics Committees of the Chinese University of Hong Kong, and all participants gave written informed consent (CREC 2019-080). Referral sources included hospital-based specialty clinics, community clinics, and private general practitioners(1). Between 1994 and 2007, 10,129 patients were enrolled, among whom 6,018 had genotype data of good quality. Among these individuals, one with non-type 2 diabetes, two with unknown types of diabetes, four with missing information on age of diagnosis were excluded. We finally included 6,011 individuals in the analysis (Figure S1). The censored date is 31 December 2019.

***Hong Kong Diabetes Biobank (HKDB)***

Hong Kong Diabetes Biobank (HKDB) is an ongoing prospective cohort initiated in 2014 which has enrolled participants from 11 diabetes centers at major public hospitals across Hong Kong. Similar to HKDR, patients enrolled in HKDB received structured assessment with fasting blood samples collected for biomedical research(2). Ethical approval was obtained from the Clinical Research Ethics Committees of the Chinese University of Hong Kong and all participants gave written informed consent (CREC 2019-080). In this study, the censored date is 31 December 2019. Between 2014 and 2019, 22,222 patients were enrolled, among them 15,838 individuals had genotype data of good quality. After excluding 1,077 overlapped with those enrolled in HKDR, 423 with type 1 diabetes, 957 with unknown types of diabetes, 35 with non-Chinese ethnicity, 919 with unknown ethnicities, 840 with missing data on age at diagnosis, we finally included 13,176 individuals with type 2 diabetes.

***PRISM***

The Precision Medicine to Redefine Insulin Secretion and Monogenic Diabetes-Randomised Controlled Trial (PRISM-RCT) enrolled Chinese with non-type 1 diabetes diagnosed at age ≤40 years(3). Participants were referred from hospital-based specialist clinics, hospital-based family medicine clinics and community-based general outpatient clinics in the New Territories East Cluster (CREC 2019-080). At study enrolment, all participants underwent structured metabolic and complication assessment. Blood samples were collected for biogenetic tests and genetic analysis. After enrolment, participants were assigned to either precision multidisciplinary care or usual care to evaluate whether multidisciplinary care guided by individual biogenetic characteristics can improve metabolic indices and reduce the risk of diabetes complications (Registered with ClinicalTrials.gov, NCT04049149). We included 873 individuals with genotyping data passing quality control as an additional validation cohort for PRS analysis.

***Shanghai Nicheng Cohort***

Shanghai Nicheng cohort was a community-based and prospective cohort recruited between April 2013 and August 2014(4). Among 17,212 participants who completed the baseline survey, 11,021 participants were randomly selected for subsequent genotyping and analysis with 2,551 of them diagnosed as T2D. Among these participants, 4,828 participated in the follow-up study from May to September 2018. This study was conducted in accordance with the principles of the Second Revision of the Declaration of Helsinki. All participants have provided written informed consent. This study was also approved by the institutional review board of the Shanghai Jiao Tong University Affiliated Sixth People’s Hospital (Approval number: 2015-KY-002(T)).

***“Better Health for Better Hong Kong” (BHBHK) Cohort***

Individuals without known history of diabetes were recruited from a community-based cohort, the “Better Health for Better Hong Kong” (BHBHK). The latter was established during a territory‐wide health promotion and screening campaign targeting the workforce with income close to or below the median salary in Hong Kong. Participants were randomly selected according to the distribution of occupational groups(5). A subset of participants was randomly selected to undergo detailed clinical and laboratory assessment with written informed consent at the Prince of Wales Hospital (PWH) in 1998-2003. They were recalled in 2010-2014 for ascertainment of glycemic status. We included 853 subjects without diabetes at baseline and at follow-up who had genotyping data with good quality in this study (Figure S1). Ethical approval was obtained for genetic analysis of these community-based and clinic-based cohorts (CREC 2019-080).

**Baseline Assessments**

All individuals with diabetes from Hong Kong underwent structured assessment at baseline including demographic status, medical history, clinical measurements and laboratory tests after an 8 hour overnight fast. All individuals eligible for analysis had measurements of fasting plasma glucose (FPG), HbA1c, lipid profile (fasting total cholesterol [TC], triglycerides [TG], high-density lipoprotein-cholesterol [HDL‐C] and calculated low-density lipoprotein-cholesterol [LDL‐C]), estimated glomerular filtration rate (eGFR) and random spot urinary albumin: creatinine ratio (ACR).

In Shanghai Nicheng cohort, individuals’ medical history, anthropometric indices and biochemical traits were recorded. Metabolic indexes including glycated hemoglobin A1c (HbA1c), serum creatinine, total cholesterol (TC), triglyceride (TG), high-density lipoprotein cholesterol (HDL-c), low-density lipoprotein cholesterol (LDL-c) and free fatty acids level were measured in blood samples. Subjects without a self-reported history of diabetes underwent 75-gram oral glucose tolerance test (OGTT) when plasma glucose and insulin levels at 0, 30, 120 minutes were measured. Diabetes was diagnosed according to the 2010 criteria of the American Diabetes Association (ADA)(6). Young-onset diabetes (YOD) is defined as diagnosis of diabetes before the age of 40 years. The EPI formula was used to estimate glomerular filtration rate (eGFR) (7). Estimated GFR less than 60 ml/min/1.73m² and/or random urine albumin-to-creatinine ratio (ACR) of 30 mg/g or more (or 3 mg/mmol) was diagnosed as chronic kidney disease (CKD).

**Genotyping, Quality Control (QC) and Imputation**

The genotyping arrays for Hong Kong cohorts were shown in below table:

| Cohorts | Disease status | Sample size included in analysis | Genotyping platform |
| --- | --- | --- | --- |
| HKDR | T2D | 6011 | Illumina Omni2.5+Exome Array |
| HKDB-P1 | T2D | 5987 | Infinium® Global Screening Array |
| HKDB-P2 | T2D | 5326 | Infinium® Asian Screening Array |
| HKDB-P3 | T2D | 1863 | Infinium® Asian Screening Array |
| PRISM | T2D | 873 | Infinium® Asian Screening Array |
| BHBHK | non-diabetes | 332 | Illumina Omni2.5+Exome Array |
|  | non-diabetes | 320 | Infinium® Asian Screening Array |

In cohorts from Hong Kong, the per-individual QC of genotype data consists of four steps: (1) sex-checking based on the genotype call from chromosome X; (2) detection of low-quality samples based on call rate and heterozygosity rate; (3) detection of possible familial relationship or duplicated individuals using estimates of identity-by-descent (IBD); (4) detection of population stratification by performing principal component (PC) analysis. Only biallelic autosomal SNPs were included in the per-marker QC. SNPs were excluded from further analysis if (1) Hardy–Weinberg equilibrium (HWE) p <1×10^−4^ and (2) minor allele frequency (MAF) < 1%; or (3) call rate < 95%. In particular, SNPs with MAF ≥ 1% but ≤ 5% were excluded if their call rate was < 99%. Within each individual cohort, we imputed the genotype data to the 1000 Genomes Project phase III reference panel (October 2014) using the Michigan Imputation Server. The genome build of genotype data from PRISM was transferred from assembly GRCh38 to GRCh37 by liftOver tool (<https://genome.ucsc.edu/cgi-bin/hgLiftOver>) before imputation(8). Imputed SNPs with MAF < 1% or imputation quality score r^2^ < 0.5 were removed.

In Shanghai Nicheng cohort, genomic DNA was extracted from whole blood samples using QIAamp DNA Blood Mini Kit (Hilden, Germany). Genotyping was performed with Infinium Asian Screening Array and Multi-Ethnic Global Array platform (Illumina, San Diego, USA). Quality control for the SNPs included Hardy-Weinberg equilibrium (P>0.01), concordant rate>99% and call rate>98%. Sample call rate for individuals was >95%.

**Functional Annotations of Variants**

We used the dbSNP database (<https://www.ncbi.nlm.nih.gov/snp/>) to annotate SNPs and the NCBI Gene database (<https://www.ncbi.nlm.nih.gov/gene>) to annotate gene information(9). We used the GTEx project database (<https://www.gtexportal.org/home/>) to explore associations of top signals with expression quantitative trait loci (eQTL)(10). We used online DAVID Functional Annotation Bioinformatics Microarray Analysis (<https://davidbioinformatics.nih.gov/>) to perform gene set and pathway enrichment analyses(11, 12). We extracted published GWAS summary statistics for glycemic and other metabolic traits from the Type 2 Diabetes Knowledge Portal (<https://t2d.hugeamp.org/>)(13, 14).

**Heritability and genetic correlation estimation**

We used LD Score regression (LDSC) to estimate SNP-based heritability for age at diagnosis (ADD) and YOD in the combined discovery and internal replication cohort (HKDR, HKDB-P1, HKDB-P2, and HKDB-P3)(15). Precalculated LD scores for East Asian population were downloaded from the resources provided by LDSC. Identifiers for variants (RS number) were annotated with the reference from SNP database dbSNP157 from NCBI (<https://www.ncbi.nlm.nih.gov/snp/>). Genetic correlation between ADD and YOD was estimated by LDSC(16). Given that LD estimation of SNPs and sample overlapping between GWAS of different traits will influence the accuracy of statistical estimation for genetic correlation, we split the discovery cohort (HKDR and HKDB-P1) and internal replication cohort (HKDB-P2) to perform meta-GWAS for ADD and YOD respectively, and used them as two sets of GWAS summary statistics to estimate the genetic correlation between ADD and YOD.

**Calculations of Variables**

In the BHBHK cohort, we used five timepoint plasma glucose and insulin during OGTT to estimate beta-cell function and insulin sensitivity. Insulinogenic index and disposition index were calculated by the formula below:

Insulinogenic index $=\frac{Ins30-Ins0}{Gluc30-Gluc0}$

Disposition index: $DI=\frac{Insulinogenic index\div6}{HOMA IR}$

Matsuda index of whole-body insulin sensitivity:

$$ISI=\frac{10000}{\sqrt{Gluc0\times Ins0\times mgluogtt\times minsogtt\div36}}$$

where Ins0 and Ins30 are plasma insulin levels at 0 and 30 minutes; Gluc0 and Gluc30 are plasma glucose (PG) levels at 0 and 30 minutes; mgluogtt is the average of PG at 0, 30, 60, 90 and 120 minutes, minsogtt is the average of plasma insulin at 0, 30, 60, 90 and 120 minutes during 75-gram OGTT. Units for insulin and glucose are pmol/L and mmol/L respectively.

In the diabetes cohort consisting of HKDR, HKDB and PRISM, we limited our analysis to participants with FPG of 3–25 mmol/L and fasting CP level of 0.2–3.5 nmol/L for using the Homeostasis Model Assessment (HOMA2) calculator v2.2.3 (<https://www.dtu.ox.ac.uk/homacalculator/>)(17). Amongst individuals from the HKDR and PRISM cohort with fasting CP, we estimate the HOMA2 of beta-cell function (HOMA2-%B) and insulin resistance (HOMA2-IR) for analysis with top signals and Polygenic risk scores (PRS) in HKDR and PRISM cohorts.

**Statistical Analysis**

To minimize batch effects and technical heterogeneity, we looked up the top variants from meta-GWAS for their associations with YOD compared with non-diabetes in two batches respectively: (1) 1320 YOD from HKDR versus 332 non-diabetes individuals from BHBHK, genotyped by Illumina Omni2.5+Exome Array; (2) 1029 YOD from HKDB-P2 versus 320 non-diabetes individuals from BHBHK, genotyped by Infinium® Asian Screening Array. We performed meta-analysis on the look-up results from these individual datasets.

We used genome-wide significant SNPs from this study to calculate genetic risk score (GRS). We used beta-coefficient for each SNP from the overall meta-GWAS from stage one (HKDR, HKDB-P1 and HKDB-P2) to compute GRS with validation in HKDB-P3 and PRISM cohorts. Details of effect alleles and their weights are shown in Table S19. In analysis conditioned on published PRS, we used the risk allele and beta-coefficient of each SNP reported by the published GWAS for (1) ADD in European, South Asian and trans-ethnic group (Table S1), and (2) type 2 diabetes in East Asian populations to compute the PRS in our Chinese cohorts (Table S2).

In individuals without diabetes, we examined the associations of top variants and ADD-GRS with glycemic traits. These included 1-hour PG, insulinogenic index, disposition index, and Matsuda index derived from 75g OGTT results. Models were adjusted for baseline age, sex and the first four PCs.

In individuals with diabetes, we examined the associations of top variants and ADD-GRS with glycemic traits at baseline. These included HbA1c, fasting CP, HOMA2-%B and HOMA2-IR. Since CP and HOMA2 indexes are only available in HKDR and PRISM cohorts, we performed meta-analyses for these three traits in the aforementioned two cohorts. Main models were adjusted for baseline age, sex and the first four PCs, whereas model 2 was additionally adjusted for BMI and use of oral glucose lowering drugs and insulin at assessment to evaluate the independent effect of the top variants and ADD-GRS on the associations with glycemic traits in diabetes. In all analyses, we calculated PCs for individuals in each cohort (HKDR, HKDB-P1, HKDB-P2, HKDB-P3, PRISM and LKS), and added the first four PCs as covariates in GWAS.

Population attributable fraction (PAF) of the genetic variant for YOD was calculated using risk ratio (RR) estimated from Poisson regression with robust standard errors (SE) and under additive genetic model(18). We first performed Poisson regression in each cohort of HKDR, HKDB-P1 and HKDB-P2 with the formula:

log(*E*[*Y*]) = *α_k_* + *β_k_*d + *γ*X

where d is the imputed dosage data of genotypes (0-2), X is covariates included in the regression model (sex, BMI, the first four PCs). The per-allele *RR_k_* was obtained from exp(*β_k_*). Then we combined *β_k_* from each individual cohort by random-effects model to obtain *RR_meta_* from exp(*β_meta_*). We estimated the risk-allele frequency in each cohort as $ṗ_{k}= \frac{đ_{k}}{2}$, where đ_k_ is the mean dosage of the individual cohort. We combined individual frequencies into a single frequency by weighing the number of individuals with T2D in each cohort as: $ṗ= \frac{\sum_{k} n_{k}ṗ_{k}}{\sum_{k} n_{k}}$. Under the Hardy–Weinberg equilibrium (HWE), we estimated genotype frequencies as: p_0_ = (1-ṗ)^2^, p_1_ = 2ṗ(1-ṗ), p_2_ = ṗ^2^. With the corresponding RR for genotype 0/1: *RR_1_* = *RR_meta_*, and for genotype 1/1: *RR_2_* = *RR*^2^_meta_, we calculated RR-based PAF as:

$$PAF= \frac{p_{1}\left( {RR}_{1}-1 \right)+p_{2}({RR}_{2}-1)}{1+p_{1}\left( {RR}_{1}-1 \right)+p_{2}({RR}_{2}-1)}$$

**SUPPLEMENTARY FIGURES:**

**Supplementary Figure 1. Study participants and selection flow**

HKDR (n=10129)

BHBHK (n=863)

Shanghai Nicheng Cohort with genotyping data(n=11,021)

PRISM (n=883)

With available genotype data

With available genotype data

HKDB (n=22222)

With available genotype data

BHBHK (n=652)

HKDR (n=6018)

HKDB Phase 3 (n=1986)

HKDB Phase 1 (n=7037)

HKDB Phase 2 (n=6548)

Excluded:

a. Non-Chinese

b. Type 1 diabetes / unknown types of diabetes

c. Overlapped samples

d. Missing data on age of diagnosis

e. Samples failing quality control / withdrawal

PRISM:

Data on ADD: 873

Shanghai Nicheng Cohort (n=2551)

a. data on ADD: 2551

b. YOD vs. LOD:

52 vs. 2499

HKDB Phase 3 (n=1863)

a. data on ADD: 1863

b. YOD vs. LOD:

299 vs. 1564

b. Lean YOD vs. Obese LOD:

47 vs. 917

HKDB Phase 2 (n=5326)

a. Data on ADD: 5326

b. YOD vs. LOD:

1029 vs. 4297

c. YOD vs. Healthy CTRL:

1029 vs. 320

d. Lean YOD vs. Obese LOD:

179 vs. 2481

HKDB Phase 1 (n=5987)

a. Data on ADD : 5987

b. YOD vs. LOD:

1002 vs. 4985

c. Lean YOD vs. Obese LOD:

200 vs. 275

HKDR (n=6011)

a. Data on ADD: 6011

b. YOD vs. LOD:

1320 vs. 4691

c. YOD vs. Healthy CTRL:

1320 vs. 332

d. Lean YOD vs. Obese LOD:

392 vs. 2207

**Supplementary Figure 2. Manhattan plots, Q-Q plots and regional plot for the top variant of genome-wide association studies (GWAS) of young-onset diabetes (YOD) versus later-onset diabetes (LOD) in discovery cohorts (HKDR, HKDB-P1) (A, B), and in combined discovery (HKDR, HKDB-P1) and internal replication cohort (HKDB-P2) (C, D).**

Abbreviations: *PADI1*, peptidyl arginine deiminase 1; *TARS2P1*, threonyl-tRNA synthetase 2 pseudogene 1.

**Supplementary Figure 3. Manhattan plots of genome-wide associations analysis (GWAS) of age at diabetes diagnosis (ADD) conditioning on PRS for age at diabetes diagnosis from trans-ancestry (A), South Asians (B), Europeans (C), and East Asian T2D PRS (D).**

Models were adjusted for sex, BMI, and the first four PCs. Abbreviations: *CDKAL1*, CDK5 regulatory subunit associated protein 1-like1; *KCNQ1*, potassium voltage-gated channel subfamily Q member 1; *PAX4*, paired box 4.

**Supplementary Figure 4. Correlation matrix between T2D pathway-specific PRS and ADD-GRS in the diabetes cohorts (HKDR, HKDB-P1, HKDB-P2) (T2D) and non-diabetes BHBHK cohort (Control).**

*, Bonferroni corrected *p*<0.05.

**Supplementary Figure 5. Manhattan plots and Q-Q plots of genome-wide association studies (GWAS) of age at diabetes diagnosis (ADD) in men (A) and women (B), and of young-onset diabetes (YOD) versus later-onset diabetes (LOD) in men (C) and women (D) in discovery cohort.**

Abbreviations: *OLIG3*, oligodendrocyte transcription factor 3; *CDC123*, cell division cycle 123.

**Supplementary Figure 6.** **Manhattan plots and Q-Q plots of genome-wide association studies (GWAS) of age at diabetes diagnosis (ADD) (A) young-onset diabetes (YOD) versus later-onset diabetes (LOD) (B) in discovery cohort after excluding individuals with monogenic diabetes.**

Abbreviations: *CDKAL1*, CDK5 regulatory subunit associated protein 1-like1; *KCNQ1*, potassium voltage-gated channel subfamily Q member 1; *PADI1*, peptidyl arginine deiminase 1; *PAX4*, paired box 4.

**References:**

1.	Chan JC, Lim L-L, Luk AO, Ozaki R, Kong AP, Ma RC, et al. From Hong Kong Diabetes Register to JADE Program to RAMP-DM for data-driven actions. Diabetes Care. 2019;42(11):2022-31.

2.	Jiang G, Luk AO, Tam CHT, Lau ES, Ozaki R, Chow EYK, et al. Obesity, clinical, and genetic predictors for glycemic progression in Chinese patients with type 2 diabetes: A cohort study using the Hong Kong Diabetes Register and Hong Kong Diabetes Biobank. PLoS Med. 2020;17(7):e1003209.

3.	O CK, Fan YN, Fan B, Lim C, Lau ESH, Tsoi STF, et al. Precision Medicine to Redefine Insulin Secretion and Monogenic Diabetes-Randomized Controlled Trial (PRISM-RCT) in Chinese patients with young-onset diabetes: design, methods and baseline characteristics. BMJ Open Diabetes Res Care. 2024;12(3).

4.	Chen P, Hou X, Hu G, Wei L, Jiao L, Wang H, et al. Abdominal subcutaneous adipose tissue: a favorable adipose depot for diabetes? Cardiovasc Diabetol. 2018;17(1):93.

5.	Ko G, Chan J, Chan A, Wong P, Hui S, Tong S, et al. Association between sleeping hours, working hours and obesity in Hong Kong Chinese: the ‘better health for better Hong Kong’health promotion campaign. International Journal of Obesity. 2007;31(2):254-60.

6.	Diagnosis and classification of diabetes mellitus. Diabetes Care. 2010;33 Suppl 1(Suppl 1):S62-9.

7.	Levey AS, Stevens LA, Schmid CH, Zhang YL, Castro AF, 3rd, Feldman HI, et al. A new equation to estimate glomerular filtration rate. Ann Intern Med. 2009;150(9):604-12.

8.	Hinrichs AS, Karolchik D, Baertsch R, Barber GP, Bejerano G, Clawson H, et al. The UCSC Genome Browser Database: update 2006. Nucleic Acids Res. 2006;34(Database issue):D590-8.

9.	Sayers EW, Beck J, Bolton EE, Brister JR, Chan J, Connor R, et al. Database resources of the National Center for Biotechnology Information in 2025. Nucleic Acids Res. 2025;53(D1):D20-d9.

10.	Lonsdale J, Thomas J, Salvatore M, Phillips R, Lo E, Shad S, et al. The Genotype-Tissue Expression (GTEx) project. Nature Genetics. 2013;45(6):580-5.

11.	Sherman BT, Hao M, Qiu J, Jiao X, Baseler MW, Lane HC, et al. DAVID: a web server for functional enrichment analysis and functional annotation of gene lists (2021 update). Nucleic Acids Res. 2022;50(W1):W216-w21.

12.	Huang da W, Sherman BT, Lempicki RA. Systematic and integrative analysis of large gene lists using DAVID bioinformatics resources. Nat Protoc. 2009;4(1):44-57.

13.	Costanzo MC, Roselli C, Brandes M, Duby M, Hoang Q, Jang D, et al. Cardiovascular Disease Knowledge Portal: A Community Resource for Cardiovascular Disease Research. Circ Genom Precis Med. 2023;16(6):e004181.

14.	Costanzo MC, von Grotthuss M, Massung J, Jang D, Caulkins L, Koesterer R, et al. The Type 2 Diabetes Knowledge Portal: An open access genetic resource dedicated to type 2 diabetes and related traits. Cell Metab. 2023;35(4):695-710.e6.

15.	Bulik-Sullivan BK, Loh P-R, Finucane HK, Ripke S, Yang J, Patterson N, et al. LD Score regression distinguishes confounding from polygenicity in genome-wide association studies. Nature Genetics. 2015;47(3):291-5.

16.	Bulik-Sullivan B, Finucane HK, Anttila V, Gusev A, Day FR, Loh P-R, et al. An atlas of genetic correlations across human diseases and traits. Nature Genetics. 2015;47(11):1236-41.

17.	Wallace TM, Levy JC, Matthews DR. Use and abuse of HOMA modeling. Diabetes Care. 2004;27(6):1487-95.

18.	Mansournia MA, Altman DG. Population attributable fraction. BMJ. 2018;360:k757.
